# Evaluating the impacts of climate change and land-use change on future droughts in northeast Thailand

**DOI:** 10.1038/s41598-024-59113-4

**Published:** 2024-04-28

**Authors:** Dibesh Khadka, Mukand S. Babel, Tawatchai Tingsanchali, Jessica Penny, Slobodan Djordjevic, Abayomi A. Abatan, Alessio Giardino

**Affiliations:** 1 Water Engineering and Management (WEM), School of Engineering and Technology (SET), Asian Institute of Technology (AIT), Klong Luang, P.O. Box 4, Pathum Thani, 12120 Thailand; 2 Centre for Water and Climate Adaptation (CWCA), School of Engineering and Technology (SET), Asian Institute of Technology (AIT), Klong Luang, P.O. Box 4, Pathum Thani, 12120 Thailand; 3 College of Engineering, Mathematics and Physical Sciences, University of Exeter (UoE), Exeter, UK; 4 Asian Development Bank (ADB), Manila, Philippines

**Keywords:** Climate change, Hydrology, Natural hazards

## Abstract

The impacts of climate change (CC) on droughts are well documented, but the effects of land-use change (LUC) are poorly understood. This study compares the projected individual and combined impacts of these stressors on future droughts (2021–2050), with respect to baseline (1981–2010) in one of the major tributaries of the Mekong River. LUC impacts on hydrological droughts are minimal compared to CC, with the latter expected to shorten the recurrence interval of a 20-year return period event to every 14 years. Both CC and LUC have significant impacts on agricultural droughts with heightened sensitivity. ‘Once in a Decade’ agricultural droughts will be 40% (35%) longer and 88% (87%) more severe under the CC (LUC) scenario. Under both stressors, the events occurring every 20 years will be twice as frequent. Results highlight the intensification of future droughts and the urgency for actions to mitigate/adapt to climate change and manage land use. Future policy shall holistically address agricultural water management, sustainable land use management, and crop management to cope with future droughts. We recommend developing resilient agricultural practices, enhanced water resource management strategies, and incorporating drought risk into land-use planning to mitigate the compounded impacts of CC and LUC.

## Introduction

It is unanimous that climate change (CC) and land-use change (LUC) driven by anthropogenic activities have increased risks in the water resources system in several regions across the globe and are likely to continue in the future^1^. CC has intensified extreme hydro-climatic events, and they are most likely to continue to increase the frequency and intensity of such events in the near-future period^2,3^. 2014–2023 is the warmest ten years in a 174-year observational record, with global temperature 1.19 ± 0.12 °C above the pre-industrial era and the concentration of three main greenhouse gases—carbon dioxide, methane, and nitrous oxide reaching record levels^4^. A temperature rise of 1.5 °C is highly likely to be reached/exceeded in the 2021–2040 period^5^. Global warming will change the global climate system, primarily by affecting the transport of moisture and energy through large-scale atmospheric circulation^6^. It alters the hydrological processes and various elements of the hydrological cycle (precipitation, evapotranspiration, soil moisture, groundwater, runoff, etc.). Global warming is expected to intensify the global water cycle further, exacerbate extreme events (including floods and droughts), and lead to a global redistribution of water resources at multiple temporal and spatial scales^7^. Based on a global risks perception survey, the top three risks perceived are directly linked to climate change and natural disasters^8^. By the end of this century, climate risk is projected to increase substantially by 2–4 folds^9^. In the last twenty years, global costs of extreme weather attributable to CC are estimated at USD 143 billion per year^10^.

LUC are mainly driven by a combination of anthropogenic factors such as socio-economy, politics, environment, and technology; however, they are also affected directly/indirectly by climate change through alteration of disturbance patterns and species distributions and expansion in new areas^11,12^. Climate change can also be a catalyst to promote anthropogenic drivers of LUC. A recent study shows that almost one-third of the earth's surface has undergone land use change during 1960–2019, with deforestation and agricultural expansion in the Global South and afforestation and cropland abandonment in the Global North^13^. Between 1982 and 2015, anthropogenic climate change and land use changes resulted in degradation of 5.43 million km^2^ of dryland to desertification^14^. LUC will alter interception by the canopy, surface roughness, soil properties of the watershed, albedo, and evapotranspiration, thus affecting the hydrological cycle. At the same time, CC influences the hydrological cycle by changing precipitation, evapotranspiration, soil moisture, groundwater storages, magnitude, and timing of runoff^15,16^.

Droughts are one of the major natural disasters which have severe implications for humans and the environment. During 1970–2019, droughts were responsible for the largest loss of human lives (650,000 deaths)^17^. The deficiency of precipitation results in meteorological droughts; deficiency in soil moisture results in agricultural droughts; deficiency in flow results in hydrological droughts^18^; and reduced groundwater head and gradients result in groundwater droughts^19^. Recently, the notion of ecological droughts has been introduced: shortfalls in water availability required to sustain the ecosystems^20^. Meteorological drought normally propagates down the hydrological cycle, resulting in hydrological, agricultural, and other types of drought, such as socioeconomic and ecological droughts^21^. Different drought types mostly have positive correlations and are likely to respond to the same trigger, while they may have differences in temporal and spatial scales^22^. Climate change affects drought propagation at shorter temporal scales, while human activities, including land use change, increase the response time at longer temporal scales^23^.

The frequency and area of global agricultural droughts are projected to increase in the future, especially in the northern hemisphere^24^. Under climate change, the severity and frequency of droughts is reported to increase in several regions across the globe, including in the Willamette River basin, U.S.^25^; in the upper Yangtze River basin, China^26^; Godavari River basin, India^27^; in South Korea^28,29^; in the Lower Mekong River basin, Vietnam^30^; in Lancang-Mekong River Basin^31^; in the Johor River basin, Malaysia^32^; in the Mediterranean^33^; in Europe^34^. It is univocal from these studies that climate change will aggravate future droughts globally. A rise in temperature will increase the evapo-transpiration demands, accompanied by increased temporal variabilities of precipitation (although some regions might observe increased precipitation), thus adversely affecting droughts.

In addition to CC, land-use change (LUC) affects the hydrological cycle and has implications for hydro-metrological extremes. Land surface processes affect the severities of various extreme events such as heat waves, droughts, etc.^35^. This study also found that the conversion of natural forests to cropland and pastures in the mid-latitudes increased the frequency of hot-dry summary from 1 in 10 years to 1 in 2–3 years. A study in the agricultural region of Marathwada, India, found that anthropogenic factors have at least quintupled the risk of agricultural droughts^36^. Similarly, increased sea surface temperatures due to anthropogenic activities contributed to the East African drought of 2017^37^. The combined impacts of CC and LUC cannot be considered linearly cumulative but could be considerably different than the combined sum of the individual drivers^38^. Moreover, CC and LUC impacts may also vary temporally^15^. LUC impacts can be significant for drought analysis as an alteration in water balance elements, such as evapotranspiration and soil moisture over a prolonged period, can further worsen drought conditions. Therefore, it is necessary to consider both drivers of change for assessing future drought.

**Figure 1:**
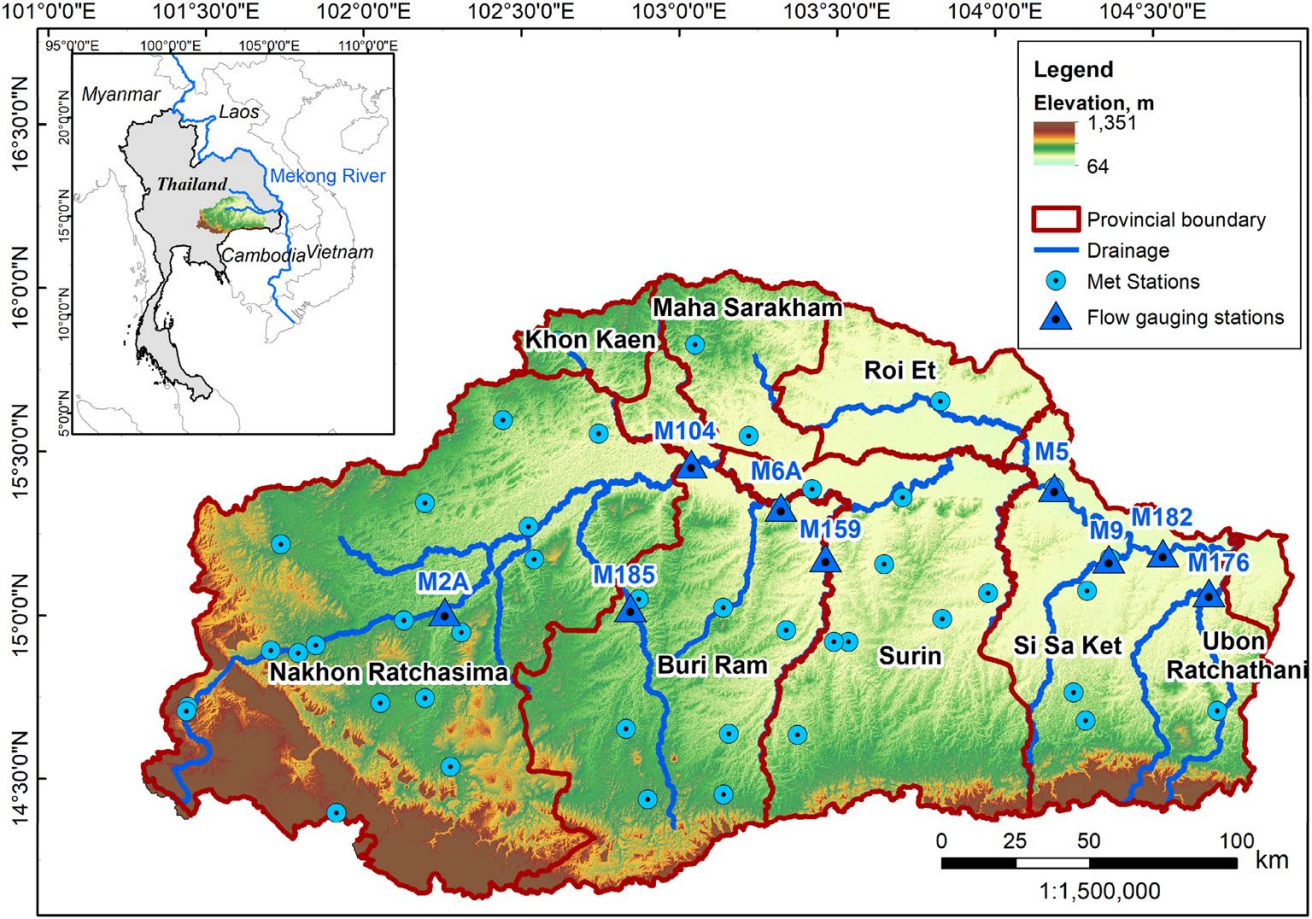
The Mun River basin in northeast Thailand. Dark red lines show provincial demarcation, blue circles are the meteorological stations, and blue triangles are the hydrological stations in the basin. The figure is created in ArcGIS Pro 3.1.0.

Although the CC impacts on droughts are well documented, the understanding of LUC impacts is limited. Few recent studies have attempted to quantify the impacts of both drivers on hydrological droughts^39,40^ and have inferred that CC is the major contributor to the projected increase in drought frequency. For the agriculture-dominated river basins, soil moisture is an important hydrological variable and a good indicator of agricultural droughts, and a significant knowledge gap exists in this domain. A study in the Mun River basin, Thailand, found that both CC and LUC will decrease soil moisture, although the former will likely increase and the latter will decrease water yield in the near future^41^. A comprehensive study of individual and combined impacts of CC and LUC on hydrological and agricultural droughts is lacking. Planners and policymakers in the water and agriculture sectors can make informed decisions to manage risk and resilience if they have access to information. Improving projections and understanding of future drought characteristics can assist in formulating suitable policies, strategies, and measures to address climate-related risks and manage land uses, even to neutralize the adverse impacts of climate change. It will also have applicability in drought-risk assessments, disaster-risk reduction, disaster preparedness, and infrastructure planning to cope with future changes. Thus, the present study is a novel attempt to fill this knowledge gap with a case study in the Mun River Basin of Thailand, a tributary of the Mekong River (Fig. 1). The climate change scenario developed using eight climate models from the sixth phase of the Coupled Model Intercomparison Project (CMIP6) for the near-future period (2021–2050) is used to assess changes in future droughts with respect to the baseline period of 1981–2010^42^.

Similarly, two land use change scenarios, namely, ‘Business As Usual’ (BAU) and ‘Combination of Forest Conservation and Urban Growth’ (CCU), developed in the previous study^43^, are considered in the study. Five future cases are analyzed to assess the individual and combined impacts of CC and LUC on agricultural and hydrological droughts. They are (a) climate change only (CC_only), (b) land-use change under BAU (LU_BAU_only), (c) Land-use change under CCU (LU_CCU_only), (d) climate change and land-use change under BAU (CC + BAU), and (e) climate change and land-use change under CCU (CC + CCU). Meteorological droughts are assessed using the Standardized Precipitation Index (SPI)^44^, hydrological droughts using the Standardized Runoff Index (SRI)^45^, and agricultural droughts using the Standardized Soil Moisture Index (SSMI)^21^ (please refer to “Materials and methods” for details on climate change scenario, land-use change scenarios, and hydrological modeling for the study basin).

## Results

### Observed drought characteristics

**Figure 2:**
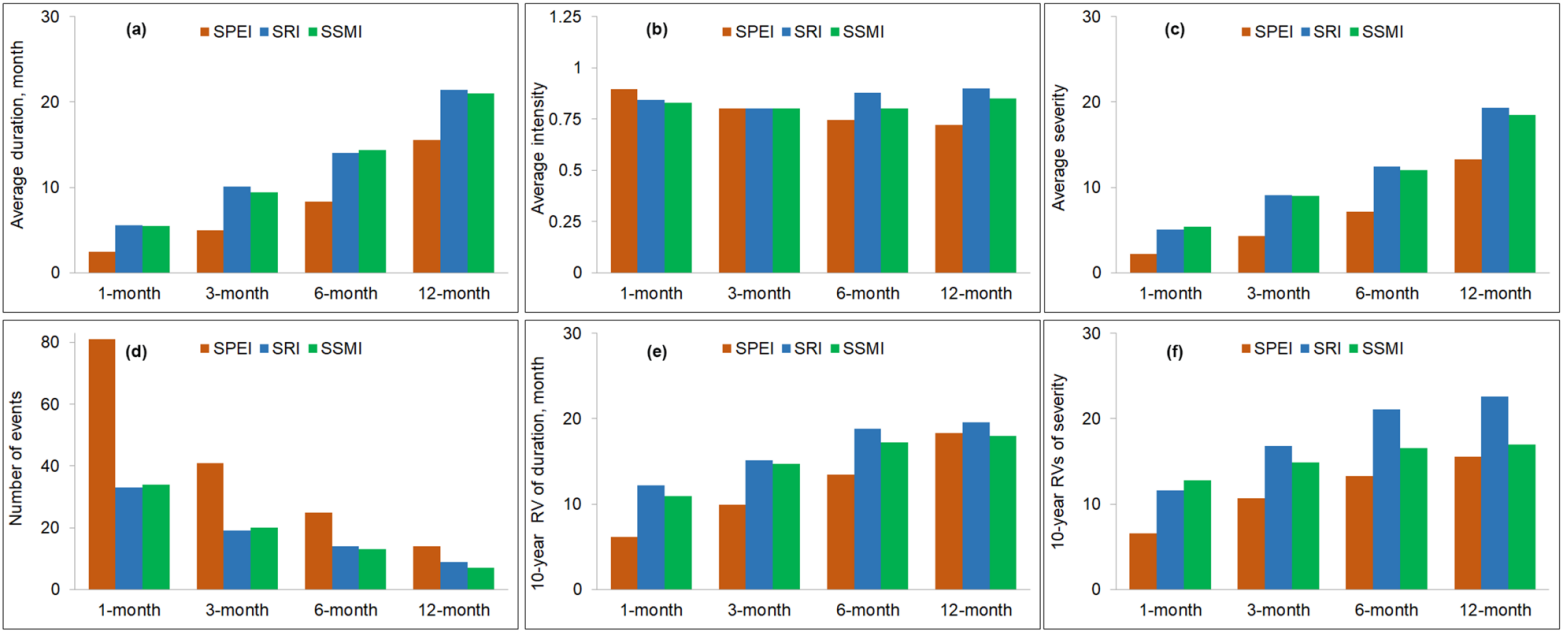
The observed drought characteristics (**a**) duration, (**b**) intensity, (**c**) severity, (**d**) the number of events, (**e**) 10-year return values (RVs) of duration, and (**f**) 10-year return values (RVs) of severity during 1981–2017 using SPEI, SRI, and SSMI. Drought intensities and severities are shown as absolute values. All sub-plots are created in Microsoft 365.

Figure 2 presents the basin-average drought characteristics during the 1981–2017 period. Among the three types of droughts, meteorological droughts have the lowest average duration and severity at all timescales. Agricultural and hydrological drought characteristics are comparable, and corresponding values for hydrological droughts are the highest among the three droughts. Meteorological droughts, when propagating to hydrological droughts, have increased in drought durations and severities, as previously reported^46^. At a 1-month timescale, the average durations of meteorological, agricultural, and hydrological droughts are 2.5, 5.5, and 5.4 months, respectively. Similarly, the three droughts have an average duration of 15.5, 21.0, and 22.0 months at a 12-month timescale, while the drought intensity is between 0.7 and 0.9. It is noted from Fig. 2d that drought events identified by SPEI are the highest and almost double those defined by SRI and SSMI, and from Fig. 2e and f, the 10-year return values for duration and severity are highest for hydrological droughts.

**Figure 3:**
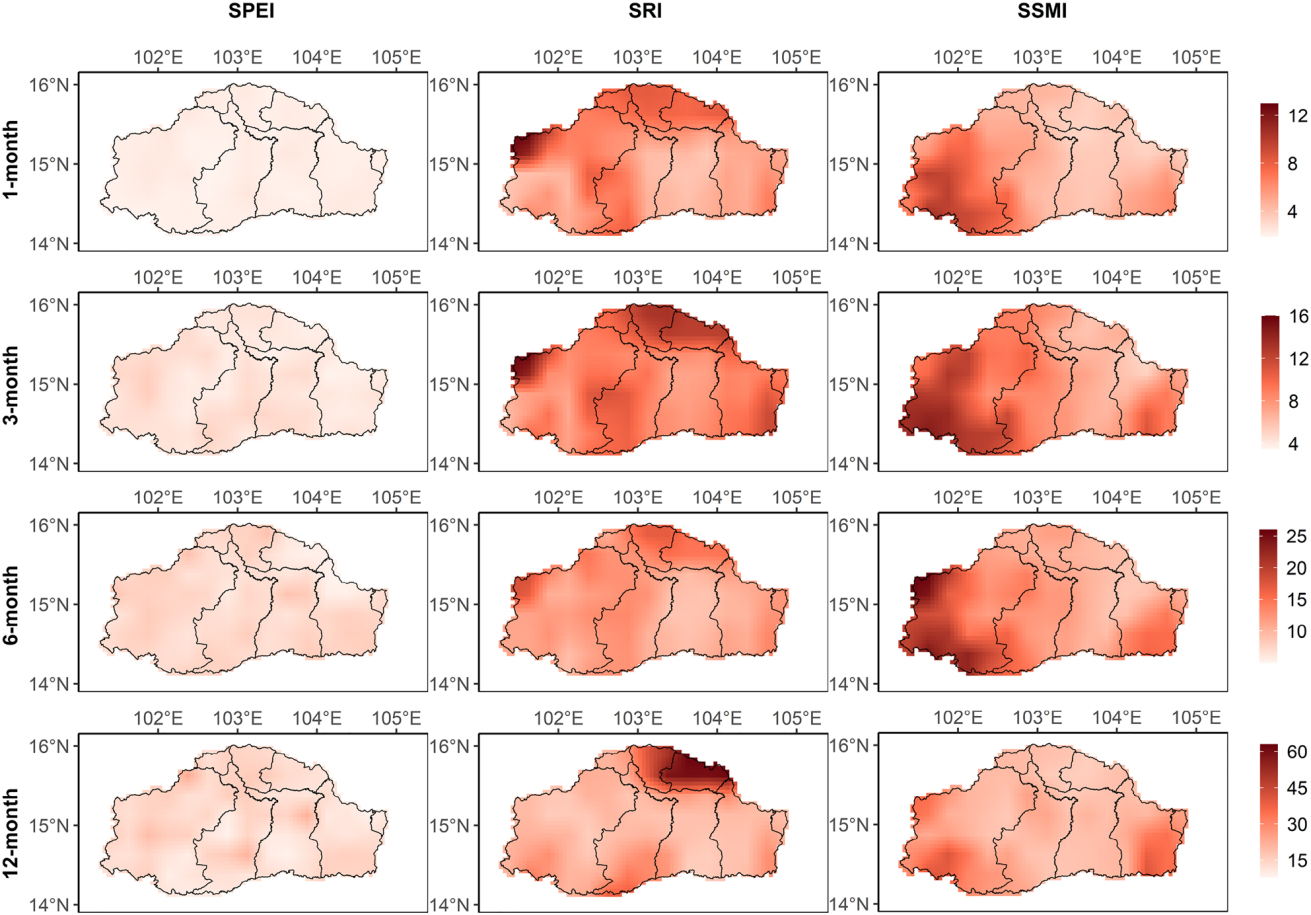
Spatial pattern of average severities (shown as absolute values) of the meteorological, hydrological, and agricultural droughts at 1-, 3-, 6-, and 12-month timescales during the baseline period. All sub-plots are created using ggplot2 library in RStudio 2022.07.2 + 576 version.

The spatial pattern of average drought severities computed using SPEI, SRI, and SSMI at four timescales overlaid over the provincial boundary is presented in Fig. 3. Compared to SRI and SSMI, magnitudes of severities for SPEI are less in the entire basin. Duration and severity of meteorological drought are higher in the central part of Nakhon Ratchasima and Buriram provinces and the southern part of Si Sa Ket province. At the same time, the northern part of the basin (Maha Sarakham and Roi Et provinces) reported higher hydrological drought severities at all timescales. Similarly, agricultural drought severities are higher in the Nakhon Ratchasima and Si Sa Ket provinces. Although meteorological and agricultural droughts show similarities in spatial patterns, differences can be expected as propagation to agricultural and hydrological droughts involves complex non-linear processes^21^.

A high degree of association among considered droughts is observed. Table 1 shows the estimated propagation time of meteorological droughts using lag correlation. The highest correlation is highlighted by bold values, which show that SRI lags SPEI by one month at a 3-month timescale and by two months at 6- and 12-month timescales. Similarly, SSMI lags SPEI by one month at 6- and 12-month timescales. It is mainly due to the lags in the basin's hydrological processes that govern the rainfall-runoff responses. An average lag time of 2 months between meteorological and hydrological droughts has been reported in the Chinese River basins^47,48^.

**Table 1:** Lag correlation of SPEI with SRI and SSMI at four timescales.

Timescale	Drought indices	Lag time (month)			
		0	1	2	3
1-month	SPEI & SRI	**0.48**	0.39	0.33	0.26
	SPEI & SSMI	**0.68**	0.44	0.29	0.21
3-month	SPEI & SRI	0.60	**0.67**	0.61	0.51
	SPEI & SSMI	**0.71**	0.71	0.59	0.41
6-month	SPEI & SRI	0.67	0.73	**0.74**	0.70
	SPEI & SSMI	0.74	**0.77**	0.75	0.69
12-month	SPEI & SRI	0.73	0.78	**0.79**	0.77
	SPEI & SSMI	0.77	**0.79**	0.78	0.76

Bold values represent the highest correlation.

### Climate change and droughts

**Figure 4:**
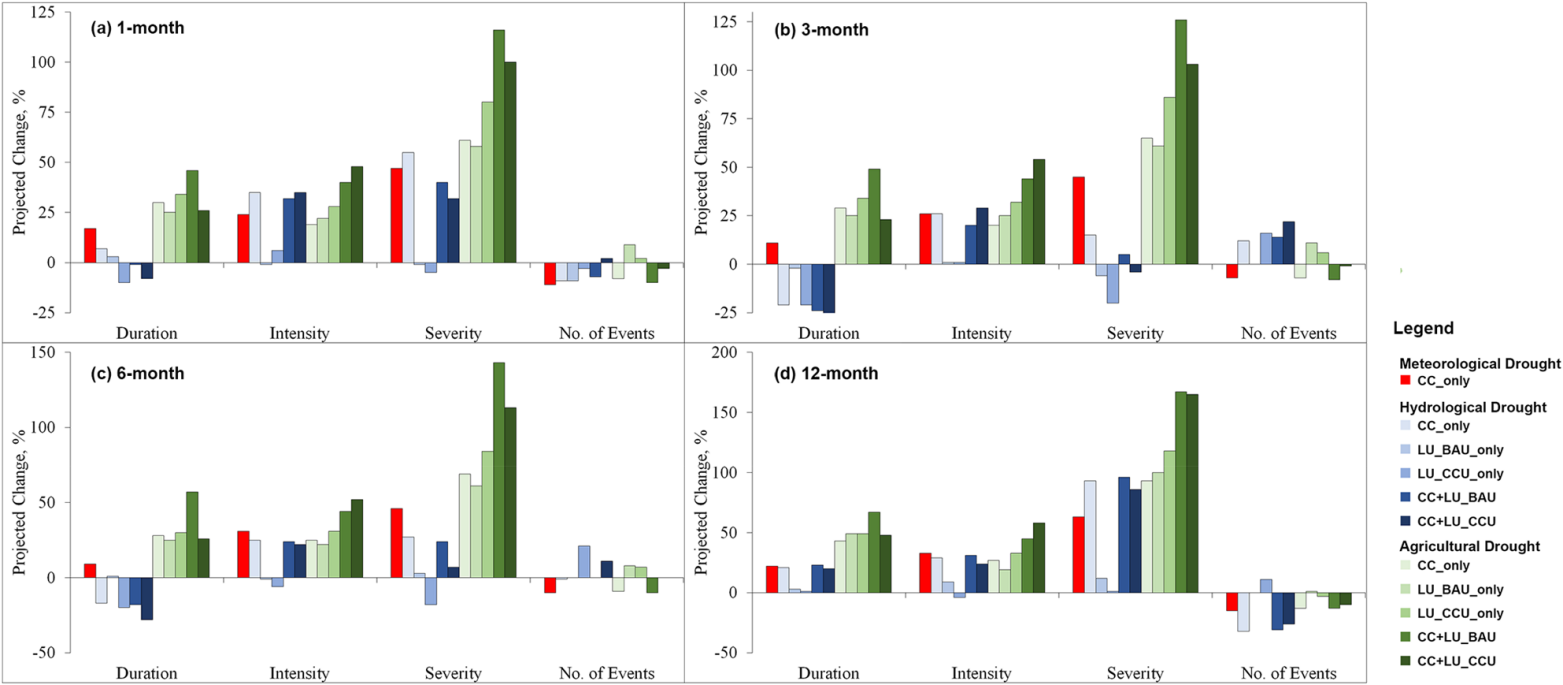
Expected changes in the drought characteristics (duration, intensity, severity, and the number of events) at four timescales and five cases ((i) climate change only (CC_only), (ii) land-use change under BAU (LU_BAU_only), (iii) Land-use change under CCU (LU_CCU_only), (iv) climate change + land-use change under BAU (CC + LU_BAU), and (v) climate change + land-use change under CCU (CC + LU_CCU) ) for the near-future period relative to baseline. All sub-plots are created in Microsoft 365.

Projected meteorological drought characteristics (multi-model ensemble) under the CC scenario are shown in Fig. 4 (shown by the red bar). Compared to the baseline, the average meteorological drought duration, intensity, and severity across various timescales will increase in the near-future by 9–22%, 24–33%, and 45–63%, respectively. However, the meteorological drought numbers will decrease by 7–15%. The maximum increase in the meteorological drought characteristics is projected at a longer timescale (12-month). Although there are some variations among the projections (shown using IQR in Supplementary Fig. S1) by individual GCMs, all climate models suggest an increase in the meteorological drought intensity, and seven out of eight models project an increase in meteorological drought severity in the near-future. The increase in duration and severity of meteorological droughts are also reflected in the higher magnitudes of their 10-year RVs (increases of 21–28% for the duration and 55–74% for the severity of the meteorological drought). The total number of events will decrease with the increase in the meteorological drought durations.

Under CC, Fig. 4 (with the light shade of blue) shows that hydrological drought durations are expected to increase for 1-month and 12-month timescales (by 7% and 21%, respectively), while it is projected to decrease at intermediate timescales of 3-month and 6-month (by 17% and 21% respectively). However, seven of eight climate models suggest that hydrological drought intensities will increase across all timescales between 25 and 35%, and severities will increase between 15 and 90%. The highest increments in drought severities will be observed at 12-month timescales. CC will also worsen the agricultural droughts as duration is expected to increase by 29–43%, average intensity by 19–27%, and average severity by 61–93% (Fig. 4, shown by the light shades of green). Increases projected for average durations and severities of agricultural droughts are the highest among the drought types considered in the study. The findings agree well with the previous study in the Be River basin, Vietnam, which estimated that agricultural drought duration could increase by up to 168% and intensity by 45% towards the end of the century^49^.

Inter-model Uncertainty (IMU) for meteorological drought severity is 35%, ranging from 15% for 1-month to 65% for 12-month timescales. The IMU is 11% for drought intensity, with a range from 5 to 15%. The smaller IMU for drought intensity indicates a stronger consensus among the climate models. Conversely, a higher IMU for severity points to more significant uncertainty in the models' projections of drought duration, as severity combines both duration and intensity. In the case of hydrological droughts, the IMU for severity stands at approximately 77%, ranging from 59% for 1-month to 120% for 12-month timescales. This suggests that the model disagreement is more pronounced at the 12-month timescale than the average projected change. The IMU for intensity is 22% (ranging from 16 to 30%), indicating a relatively better agreement. The additional layer of uncertainty introduced by hydrological modeling results in a higher IMU for drought severity in hydrological droughts compared to meteorological droughts. The larger spread indicates significant variances in model projections.

For agricultural droughts, the IMU for severity ranges from 46% for 1-month to 118% for 12-month timescales, while for intensity, it is between 7 and 19% for the respective timescales. These findings suggest a consensus among models that drought intensity will increase in the future. Although most climate models project an increase in severity, the uncertainty related to model projections is higher. It is also worth noting that the magnitude of uncertainty increases with the time scale. Compared to meteorological droughts, a higher degree of uncertainty is found for hydrological and agricultural droughts. This was also presented in the recent study^50^, which shows uncertainty associated with agricultural droughts due to climate change is up to sevenfold greater than that for meteorological droughts.

### Landuse change and droughts

LUCs are not expected to have significant impacts on hydrological droughts. Under the BAU scenario, changes in the hydrological drought duration are less than ± 3%. In contrast, severities of hydrological droughts are projected to decrease slightly at 1- and 3-month timescales while increasing by 12% for the 12-month timescale. LUC under the CCU scenario may even have positive impacts on hydrological droughts as the hydrological drought durations and severities are projected to reduce between − 10% to − 21% and − 5% to − 20%, respectively, for 1- to 9-month timescales and no changes are expected for the 12-month timescale. On the contrary, LUC will have significant impacts on agricultural droughts (Fig. 4, shown by the light shades of green). Agricultural drought durations are expected to increase by 25–50%; intensity by 19–25%; and severity by 58–100% for various timescales under BAU scenarios, while the corresponding agricultural drought characteristics will increase by 30–50%, 28–33% and 80–118% under CCU scenario. Projected rises in the agricultural drought severities increase with the timescale. These results clearly show the impacts of LUC on agricultural droughts are comparable to or even more severe than the CC in some instances.

### Climate + land-use changes and droughts

**Figure 5:**
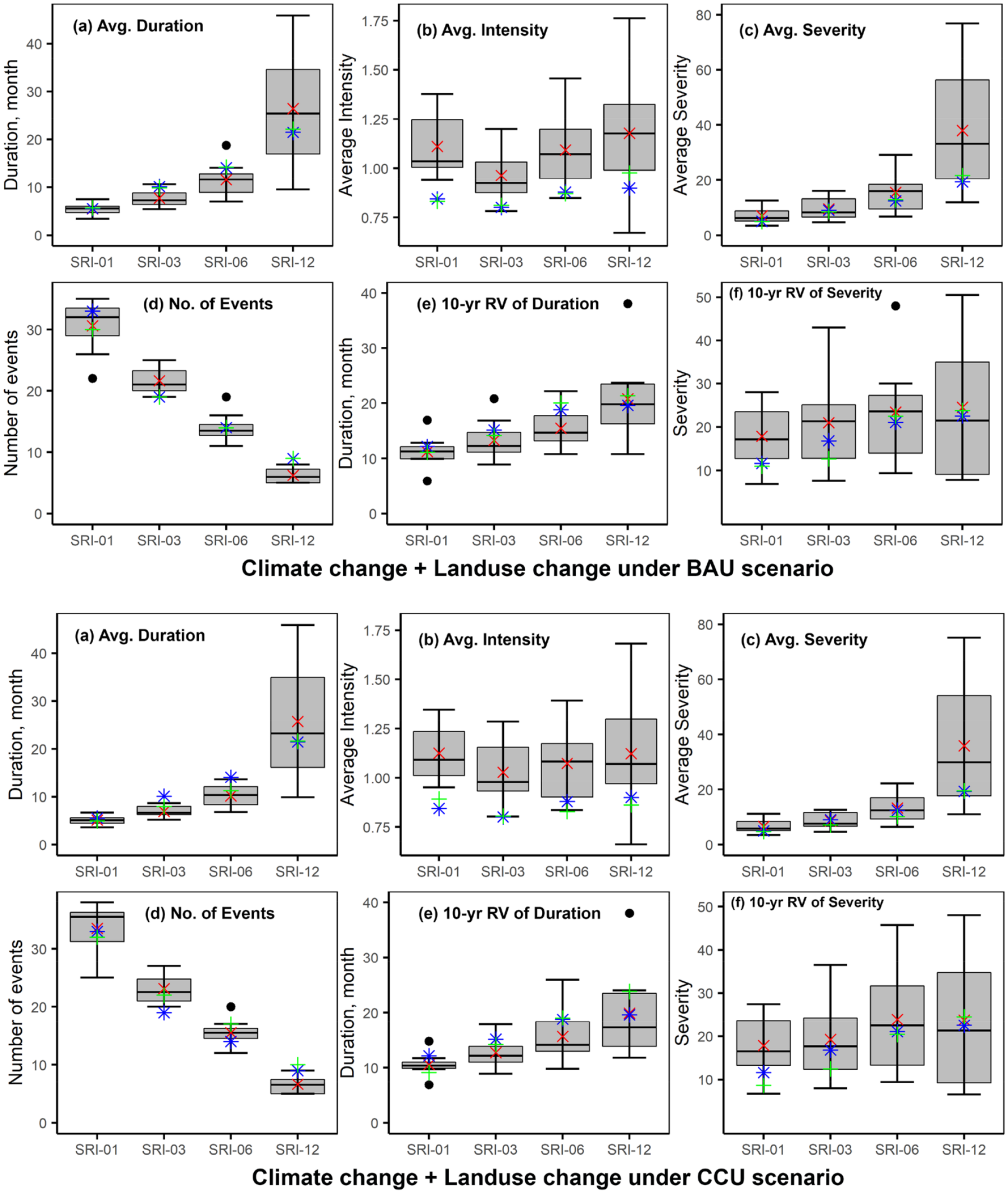
Boxplot shows the projected hydrological drought characteristics for the near future for a combined case of CC and LUC under BAU (top) and a combined case of CC and LUC under CCU (bottom). The grey boxes show IQR; the horizontal line within the box corresponds to the median value, and the whiskers show either the maximum/minimum values or 1.5 times the IQR. The red crosses are the values for the ensemble average, the green plus sign is the value considering land-use only, and the blue stars are the values during the baseline period. Severities are shown as absolute values. All sub-plots are created using ggplot2 library in RStudio 2022.07.2 + 576 version.

Under both combined cases of CC and LUC scenarios, (CC + LU_BAU) and (CC + LU_CCU), projected changes in the hydrological drought’s duration and severity are smaller than in the case considering CC only. The results are apparent in the case of CC + LU_CCU, where projected changes in hydrological drought characteristics due to CC and LUC are in opposite directions. Except for the 12-month timescale, hydrological drought durations will decrease by − 8% to − 31%. Figure 5 presents the spread of projections of hydrological droughts between individual climate models and the ensemble average. It clearly shows that when CC and LUC are considered concurrently, hydrological drought characteristics at all timescales are dominated by CC. Moreover, Fig. 5 also shows the associated uncertainties in hydrological modeling; the spread of projections resulting from individual GCMs is larger for hydrological droughts than the meteorological droughts, as shown in Fig. S1), particularly for the timescale of 12-month.

**Figure 6:**
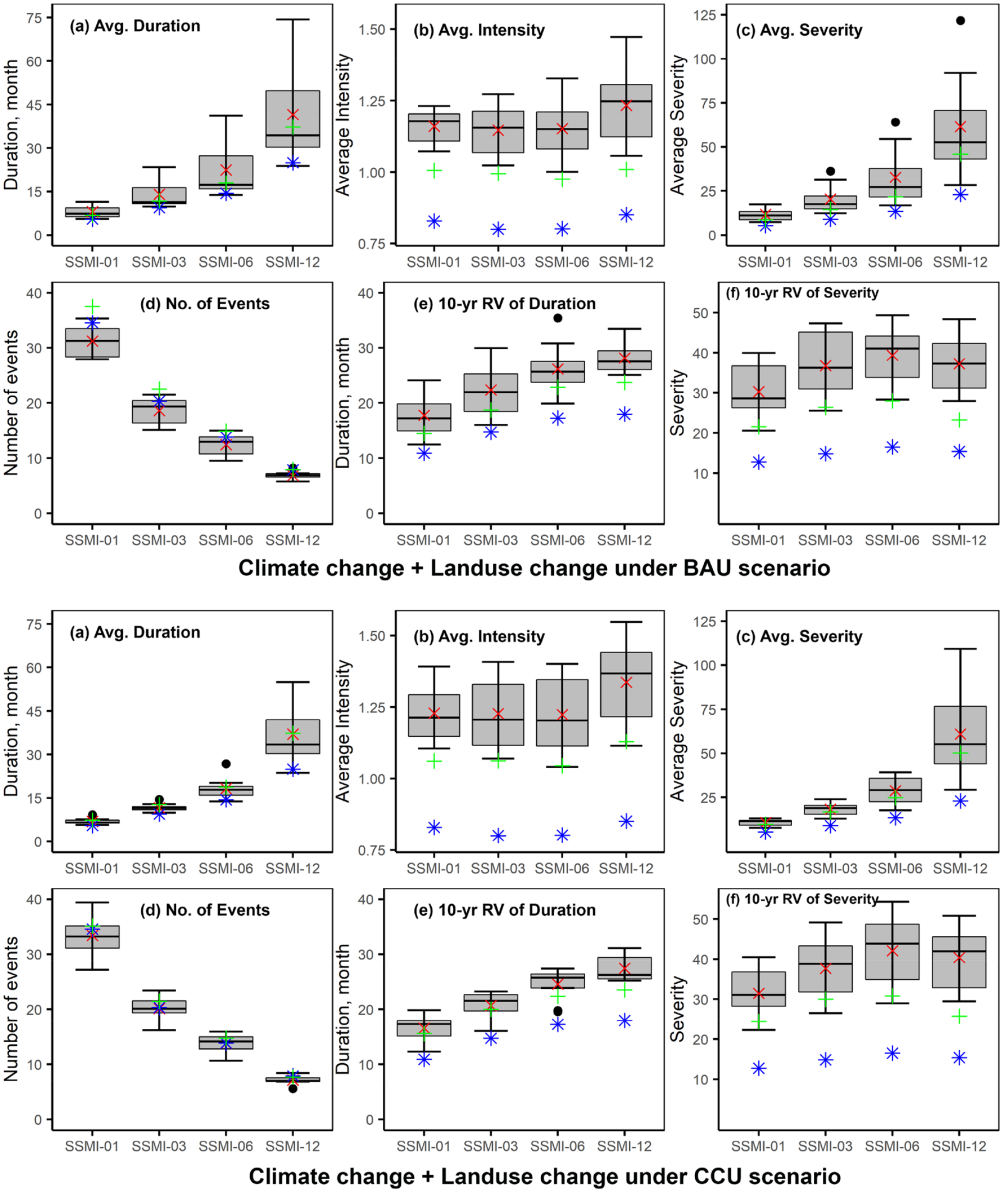
Boxplot shows the projected agricultural drought characteristics for the near future for a combined case of CC and LUC under BAU (top) and a combined case of CC and LUC under CCU (bottom). The grey boxes show IQR; the horizontal line within the box corresponds to the median value, and the whiskers show either the maximum/minimum values or 1.5 times the IQR. The red crosses are the values for the ensemble average, the green plus sign is the value considering land-use only, and the blue stars are the values during the baseline period. Severities are shown as absolute values. All sub-plots are created using ggplot2 library in RStudio 2022.07.2 + 576 version.

For agricultural droughts, the combined effects of CC and LUCs are found to have higher increases in drought durations and severities than the cases considering these stressors independently. Figure 4 (darker shades of green) shows the increase in agricultural drought durations under the combined scenario of CC and LUC for BAU (CCU) will be 45–67% (23–48%), while the agricultural drought intensities and severities will increase by 40–45% (48–58%) and 116–167% (100–165%), respectively. The range of projected changes in the agricultural drought characteristics by climate model ensemble is shown in Fig. 6. Seven out of eight models suggest increments in the agricultural drought durations, while all models agree on increments in agricultural drought severities under both combined cases of CC and LUC. The figure also indicates the marked impacts of both CC and LUC on agricultural droughts. Compared to hydrological drought, agricultural droughts will be more severe (amplified by several folds in some cases) and compared to shorter timescales (1- and 3-month), the projected increases in agricultural drought durations and severities will also be higher for the longer timescales (6-, and 12-month).

## Discussion

When meteorological droughts propagate to agricultural and hydrological droughts, they are accompanied by a reduction in the number of events and increases in the durations and severities of the agricultural and hydrological droughts. It is primarily due to the high variability of the rainfall (which is mainly the case at shorter timescales), resulting in more meteorological drought events. The variabilities in the rainfall are attenuated during the hydrological processes, resulting in less variability in soil moisture and river flows. Thus, agricultural and hydrological droughts are lesser in number but longer in duration and higher in severity than meteorological droughts. A global study also reported similar findings, which show that the spatial extent and drought characteristics increase from meteorological to hydrological and from hydrological to agricultural droughts^50^. Drought characteristics will be worse in the future due to climate change. The meteorological droughts, which occur on average once every decade, are expected to be 25% longer and 65% more severe under the climate change scenario. With the increase in temperature, evapotranspiration demands in the future are estimated to increase by 4.4% compared to the baseline. In addition, climate change will also increase the temporal variability of the rainfall in the basin by 35%, although the annual average rainfall remains more or less the same (< 1% change). As a result, future meteorological droughts are projected to be longer and more intense.

In comparison, the hydrological droughts have incongruent responses to climate change at different timescales, with future hydrological drought durations decreasing for the intermediate timescale (3- and 6-month) and increasing for the longer timescale (12-month). Results of hydrological modeling of the basin for climate change scenario show that the water yield, on average, will increase by 11%^41^. The river flows are expected to rise during May–Oct (rainy season) and reduce during Jan–Mar (dry season). However, the near-future is accompanied by an increase in temporal variabilities of river flows as the monthly and annual variabilities will increase by 18% and 41%, respectively, contributing to intensifying droughts. Compared to CC, LUC (both BAU and CCU scenarios) has less influence on future hydrological droughts as water yields (magnitude and temporal variability) are insignificantly influenced by LUC. Moreover, the LUC affects hydrological droughts opposite to CC's. Variabilities in river flows are expected to decrease by 4.4% (6.8%) for BAU (CCU) scenarios, contributing to favorable impacts on hydrological droughts. A recent study also suggests that CC may positively affect hydrological droughts due to expected changes in precipitation; however, the extreme droughts will be negatively impacted^12,51^. Once in decade hydrological droughts are expected to be 34% more severe under CC, while they will be 5% and 12% less severe under LUC of BAU and CCU, respectively. Under the combined case, they will be 21–25% more severe. These results indicate that CC is a relatively more important driver of change in hydrological droughts than LUC in the basin, which is also suggested by previous studies^39,40^. The inter-model uncertainty for hydrological drought characteristics is higher than for meteorological droughts. As rainfall-runoff transformation involves complex non-linear processes that the hydrological model does not entirely simulate, it may have added another layer of uncertainty to the results and climate model-related uncertainties.

**Figure 7:**
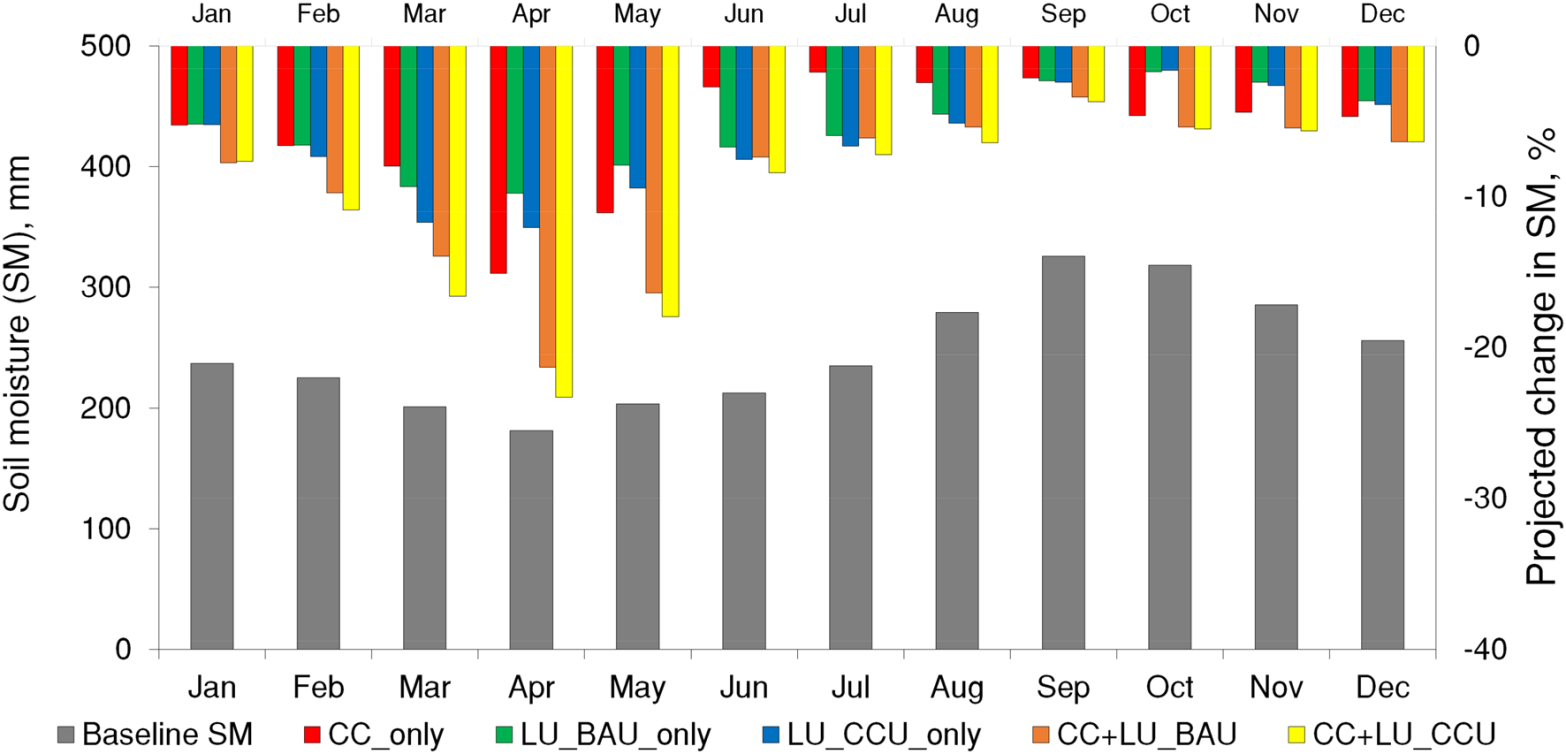
Monthly average soil moisture (SM) during baseline (left axis) and the projected change in the monthly average soil moisture under five future cases considering climate change and land-use change compared to the baseline period (right axis). The figure is created in Microsoft 365.

Most interestingly, the study finds that both CC and LUC are significant drivers of agricultural droughts in the basin. Under individual cases of CC and LUC, agricultural drought occurring once every decade will be 40% and 31–35% longer and 88% and 67–87% more severe than the baseline, respectively. With combined stressors, agricultural droughts are projected to be 50% longer and 150% more severe. A study in China^21^ also found that CC can increase agricultural drought duration by several folds. CC and LUC will affect different hydrological processes in the basin, and their combined impacts are not linearly cumulative of the individual impacts. CC predominantly alters the timing and volume of surface water converting into soil moisture, while the LUC affects the timing and amount of evapotranspiration in the basin. In the study basin, CC will increase rainfall extremes while decreasing the number of rainfall days in the near-future^42^. These will contribute to an increase in surface runoff and provide fewer opportunities for infiltration to maintain soil moisture. This reduction in future soil moisture will contribute to significant increases in agricultural droughts. A recent study in Central Asia also reported similar findings that the anthropogenic forcing was responsible for the decline in soil moisture resulting in the severity of the agricultural droughts^49^. Figure 7 shows the projected reductions in soil moisture under five future cases analyzed. Under the CC scenario, the average annual soil moisture will decrease by about 5%, while for the combined CC and LUC cases, decreases are expected to be about 8% and 9%, respectively, for BAU and CCU. On the temporal scale, soil moisture decreases are highest (close to 20%) during the summer (Mar–May) under the combined cases, while least during Sep–Nov (about 2–5%).

Further, both drivers also affect the runoff generated in the basin. In this case, even though the annual rainfall in the basin remains almost the same (< 1% change), the annual water yields will increase by about 11%. Thus, an increase in the other water balance components (evapotranspiration and runoff) will drastically affect soil moisture, resulting in higher sensitivity of the agricultural droughts to both CC and LUC.

The findings have implications in the study basin, especially as the land-use is dominated by agriculture, which is directly linked with the livelihood of most of the population. Droughts in the basin have been found to correlate well with the yields of major crops (Rice and Maize)^52^. The impacts of CC and LUC can further propagate to socioeconomic and ecological droughts. Our study has estimated CC and LUC's separate and combined impacts on future drought characteristics. It highlights the need to formulate suitable policies, strategies, and plans for developing resilience against future droughts.

## Conclusions

The research examines the future drought characteristics under climate change and land-use change scenarios using three standardized drought indices. Meteorological, hydrological, and agricultural drought characteristics are projected for 2021–2050 and compared with the baseline (1981–2010). A pivotal finding of the study is the equal significance of land use change and climate change in driving the future severity of agricultural droughts. The finding is important for understanding the multi-faceted drivers of droughts. Our study suggests that future hydrological droughts will be dictated mainly by climate change. Under climate change, once in a decade hydrological drought will be 34% more severe, whereas the impact of land-use changes could reduce their severity by up to 12%. Furthermore, agricultural droughts, for the same return period, are forecasted to worsen significantly, with an increase in severity by 88% under climate change and 67–87% due to land-use change. It is also worth noting that agricultural droughts are more sensitive to both climate change and land-use change than meteorological and hydrological droughts. The combined impacts of climate and land-use change will likely have dire repercussions on future agricultural droughts as the severities can be more than two-fold, highlighting the urgency for adaptation and mitigation strategies.

Future research can investigate the inter-linkages between land-use change, climate change, and droughts, such as how land-use changes are driven by climate change and droughts and how land-use change can influence local/ regional climate, which will further affect meteorological droughts and consequently agricultural and hydrological droughts. Land and climate have complex interactions, and the impacts of land-use on climate change have regional heterogeneity as well as origins beyond the boundary of the basin^53^. The study has considered climate-model-related uncertainties using multiple climate model outputs. However, climate scenario-related uncertainties are not considered as the future projections of climate models in HighResMIP of CMIP6 are available only for the high-emission scenario. We recommend using alternative MIPs, such as ScenarioMIP, where future projections are available for multiple SSPs to capture related uncertainties better.

## Materials and methods

### Study domain

The Mun River basin in northeast Thailand (geographically between 14.1 to 16.0°N latitudes and 101.2 to 104.9°E longitudes) is a major tributary of the Mekong River and is also significant from an agricultural perspective in the country (Fig. 1). The basin is spread over Nakhon Ratchasima, Buriram, Surin, and Si Sa Ket provinces, while partially included in Maha Sarakham, Khon Khen, Roi Et, and Ubon Ratchathani provinces. The basin has a tropical climate with three seasons. November to February is the winter season when the cool and dry winds blow westward from the South China Sea, bringing little/no rainfall (northeast monsoon). March to May is the dry season (or pre-rainy), a transition period from the northeast monsoon to the southwest monsoon^52^. June to October constitute the rainy season (associated with the southwest monsoon), when more than 80% of the annual rainfall occurs.

### Observational hydro-climatic dataset

Gridded rainfall data interpolated using the inverse distance weighing (IDW) method at 0.25-degree resolution from daily rainfall measured at 43 stations are utilized in the study^42^. The rainfall dataset was found to be consistent with several global datasets (APHRODITE, CPC, GPCC, CHIRPS). Temperature records were available only at a few stations in the basin, so Climate Prediction Center Global Land Surface Air Temperature Analysis^54^ was used. Among several global temperature products, CPC data compared better with observations, with a correlation coefficient above 0.95 and an RMSE of about 0.8 °C^42^. Observed temperature data are also interpolated to a common spatial resolution of a 0.25-degree grid using bilinear interpolation. The spatial patterns of the average of Tmax, Tmin, and rainfall during the 1981–2010 (baseline) period are presented in Supplementary Fig. S2. Limited spatial variations in Tmax and Tmin within the basin range between 32.3–33.2 °C and 22.0–22.9 °C respectively. In contrast, annual rainfall shows a significant disparity, as the western part receives an annual rainfall of about 900 mm and the eastern part about 1600 mm.

Daily river flow data for 1981–2016 were collected and measured at nine gauging stations from the Royal Irrigation Department (RID) of Thailand, as shown in Fig. 1. January to May is the low flow period in the basin, and the flow begins to increase from June and peaks during October. The annual average flows for stations M104, M5, and M182, representing the upper, middle, and lower Mun river basin, are 97, 216, and 265 m^3^/s, respectively (Supplementary Fig. S2).

### Land-use and soil dataset

Land-use maps from Thailand's Land Development Department (LDD) for the years 2000 and 2008 are utilized in this study. Agriculture is a major livelihood activity of the people, with about 75% of the total basin area under cultivation. Rice is the primary crop grown in the basin (about 55% of the entire area) (Supplementary Fig. S4), of which 90% are rainfed^55^. The next major crops grown are the Field crops, which consist of Cassava, Sugarcane, and Maize. Rice is cultivated in the middle and lower regions of the basin; field crops are mainly grown in the upper region, and the forested area is located primarily in the south. Compared to land use in 2000, the area under rice has reduced from 60.2% to 55.5%, while Perennials and orchards have increased from 1.7 to 4.9% in 2008. Soil data acquired from LDD (Supplementary Fig. S5) shows that most of the basin has sandy loamy soil with low fertility grade and a limited ability to hold water and nutrients^56^.

### Future climate projections

Future projections of Tmax, Tmin, and rainfall from prior study^42^ using eight climate models from the HighResMIPs of CMIP6 are considered. Descriptions of the models are provided in Supplementary Table S1. Future climate data are available until 2050 under the Shared Socioeconomic Pathway 5 (SSP5-8.5) scenario. In radiative forcing, the scenario is similar to RCP8.5^57^. Changes in the near-future period of 2021–2050 have been assessed relative to the baseline period of 1981–2010. Projected rainfall data for the climate model are resampled to 0.25-degree resolution using the nearest neighbor (NN) method, while bilinear interpolation is employed for temperature data. Future climate data has been corrected for biases using quantile mapping. Rainfall data is bias-corrected using empirical distribution, which avoids assumptions about distribution fitting and corrects rainfall intensity and frequency. For temperature, theoretical distribution is a better choice as it involves frequent extrapolation; hence, normal distribution is used. Details on bias corrections can be referred to Khadka et al.^42^.

The study employs equal weight methods for computing the multi-model ensemble, the justification being that climate models are considered equiprobable and the model ensemble performs better than individual models^5,58,59^. Weighting models based on their performance on specific metrics can be misleading as different metrics could potentially lead to different rankings and weights. Supplementary Table S2 shows the projected monthly changes in the considered climatic variables in the near future. The highest increase in the Tmax will occur during the Mar–May period, while for Tmin during the Nov–Dec period. Overall, increments of 1.29 °C and 1.37 °C in annual Tmax and Tmin are projected. In the case of annual rainfall, no significant changes are projected (0.5%). However, changes in temporal rainfall pattern will be observed, with increases during the rainy season by 2–8% and decreases during the dry season by 6–11%. The spatial pattern of the projected temperature changes does not show much variation within the basin (Supplementary Fig. S6), although the highest increase in Tmax is expected to be in the central parts of Nakhon. A mixed pattern will be observed for the rainfall, with projected increases in the central part and decreases in the northwest and southeast parts of the basin.

### Future land-use projections

The study has utilized two land-use change scenarios developed for the Mun River basin using the Future Land Use Simulation (FLUS) model^43^. Two LUC scenarios considered in this study are (i) Business as usual (BAU) and the Combination of forest conservation and urban growth (CCU). BAU scenario is based on the observed past land-use changes in the basin, while CCU is a multi-objective scenario combining the objectives of increased Conservation^60^ and Urban growth^61^.

Supplementary Fig. S7 shows the projected land-use in the basin for 2050 under BAU and CCU scenarios. Rice fields are projected to be reduced under both scenarios to 44.3% (in BAU) and 36.2% (in CCU). Similarly, the area under field crops, perennials & orchards, and urban areas will increase compared to 2008 LU. Field crops will increase to 21.8% and 15.6%, Perennials and Orchards will increase to 9.1% and 6.7%, and Urban will increase to 7.3% and 10.0% under BAU and CCU scenarios respectively. Forest area will decrease to 11.0% under BAU while increasing to 25% under CCU scenarios. LUC scenarios utilized in the study encapsulate the possible range of projected changes under various scenarios developed^43^.

### Soil and water assessment tool (SWAT) modeling

The hydrological modeling of the basin under baseline and near-future scenarios is conducted using the Soil and Water Assessment Tool (SWAT). It is suitable for continuous simulations in agricultural watersheds^62^. Evapotranspiration is an important hydrological process to consider for drought assessment, and SWAT is suitable as it also has a crop growth module. The watershed is divided into 57 sub-watersheds based on topography and further into 1855 Hydrologic Response Units (HRUs) comprising homogenous land-use, slope, and soil characteristics.

The model has been calibrated and validated with observed flows at nine locations, evapotranspiration (ET) (using The Global Land Evaporation Amsterdam Model, GLEAM ET^63^), and soil moisture (SM) (using the Global Land Data Assimilation System, GLDAS^64^). Calibration is carried out using the SWAT-CUP software with the Sequential Uncertainty Fitting (SUFI) algorithm, allowing sensitivity and uncertainty analyses^65^. The calibration is conducted considering the land-use map of 2008 and simulations from 2006 to 2017, and it is validated using the land use of 2000 and simulations from 1996 to 2005. P-value and sensitivity analysis are used to identify critical parameters for calibration/validation. Model performance is assessed using performance statistics such as P-factor and R-factor^65^, Nash–Sutcliffe (NS), Coefficient of determination (R^2^), and volume bias^66^. The model can reasonably simulate the hydrological process, especially the low flows^41^.

The calibrated model is applied to simulated flows and soil moisture for five future cases considering CC and LUC scenarios. They are (a) climate change only (CC_only), (b) land-use change under BAU (LU_BAU_only), (c) Land-use change under CCU (LU_CCU_only), (d) climate change + land-use change under BAU (CC + LU_BAU), and (e) climate change + land-use change under CCU (CC + LU_CCU).

### Standardized drought indices

The study has used Standardized Precipitation Evapotranspiration (SPEI)^44^ for meteorological, Standardized Runoff Index (SRI) for hydrological, and Standardized Soil Moisture Index (SSMI)^21^ for agricultural drought analyses. SPEI is a multivariate drought index that considers latent variables ( Precipitation – Potential evapotranspiration) and has been applied in several recent climate change studies^67–69^. the data is fitted to the Generalized Extreme Value (GEV) distribution^52,70^.

SRI is similar to SPEI, where flow time series data are used instead of P-PET time series, and the data is fitted into a two-parameter log-normal distribution^71,72^. SSMI is computed using the soil moisture by fitting into gamma distribution^73^. Unlike rainfall and runoff, soil moisture data may not be readily available. Following the previous research^21,30,74^, simulated soil moisture by the SWAT model is used for computing the SSMI. Drought indices are calculated at four timescales (1-, 3-, 6-, and 12-month) to assess short-term and long-term droughts. Future meteorological droughts are assessed under the case of CC only, while hydrological and agricultural droughts are assessed under five cases of CC and LUC, as discussed above.

### Characterization of droughts

A new approach^52^, has been used to identify drought events from standardized indices. Two criteria are used to define droughts: (i) drought index value less than zero for consecutive time steps, and (ii) the maximum intensity for that period is less than/or equal to – 0.5. This approach helps to omit minor drought events during analysis and prevent a single event from splitting into multiple smaller duration events, which can have consequences in drought characterization.

The univariate approach is used for drought durations and severities analyses for 10-year and 20-year return values using empirical distribution function (EDF), a non-parametric approach^75^. Future projected drought characteristics are compared with the observed period to assess changes.

### Uncertainty analysis

Inter-model uncertainty (IMU)^42,76^ related to drought intensity and severity is estimated as the standard deviation (SD) of the projected changes from the climate model ensemble given by

$$IMU=\sqrt{\frac{1}{n}\sum_{i=1}^{n}{\left({M}_{i}-\overline{M }\right)}^{2}}$$

where ‘$\overline{M }$’ is the ensemble mean projected change, and n is the number of climate models. IMU is expressed as a deviation from the ensemble average.

### Supplementary Information

Supplementary Information.

## Data Availability

Data can be accessed from the NERC EDS Environmental Information Data Centre. 10.5285/b11c040d-c3c0-43c5-a7c0-442b067dc526.
