# Supplementary material for: Evaluating the impacts of climate change and land-use change on future droughts in northeast Thailand
Source: Sci Rep. 2024 Apr 28;14:9746. doi: 10.1038/s41598-024-59113-4 (PMC11056375; doi:10.1038/s41598-024-59113-4)
Supplement: Supplementary file 1 — Supplementary Information. [file 41598_2024_59113_MOESM1_ESM.docx]

**Evaluating the impacts of climate change and land-use change on future droughts in northeast Thailand**

Dibesh Khadka^a,b^, Mukand S. Babel^a,b*^, Tawatchai Tingsanchali^a^, Jessica Penny^c^, Slobodan Djordjevic^c^, Abayomi A. Abatan^c^, Alessio Giardino^d^

^a^ Water Engineering and Management (WEM), School of Engineering and Technology (SET), Asian Institute of Technology (AIT), Thailand

^b^ Centre for Water and Climate Adaptation (CWCA), School of Engineering and Technology (SET), Asian Institute of Technology (AIT), Thailand

^c^ College of Engineering, Mathematics and Physical Sciences, University of Exeter (UoE), UK

^d^ Asian Development Bank (ADB), Manila, Philippines

***Corresponding Author:**

Mukand S. Babel

Water Engineering and Management (WEM), School of Engineering and Technology (SET), Asian Institute of Technology (AIT), Thailand

Centre for Water and Climate Adaptation, School of Engineering and Technology (SET), Asian Institute of Technology (AIT), Thailand

P.O. Box 4, Klong Luang, Pathum Thani 12120, Thailand

Email: msbabel@ait.ac.th

Tel.: +66-86-089-8949


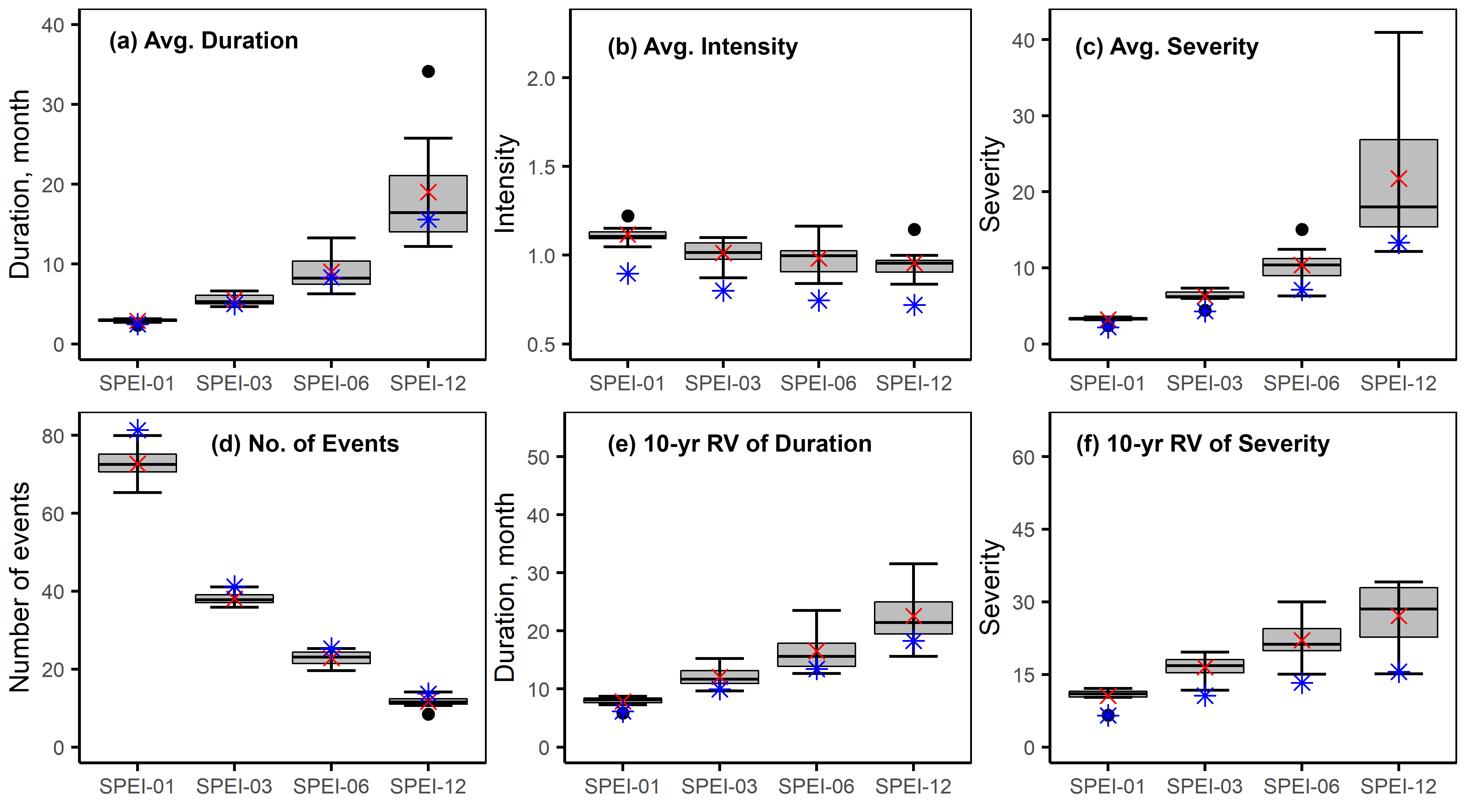


**Figure S1.** Boxplot shows the projected meteorological drought characteristics for the near future period under climate change scenario. The boxes represent the IQR with the horizontal line in the box corresponding to the median value and the whiskers to either the maximum/minimum values or 1.5 times the IQR. The red crosses are the values for the ensemble average, while the blue stars are the values during the baseline period. Severities are shown as absolute values. All sub-plots are created using ggplot2 library in RStudio 2022.07.2+576 version.


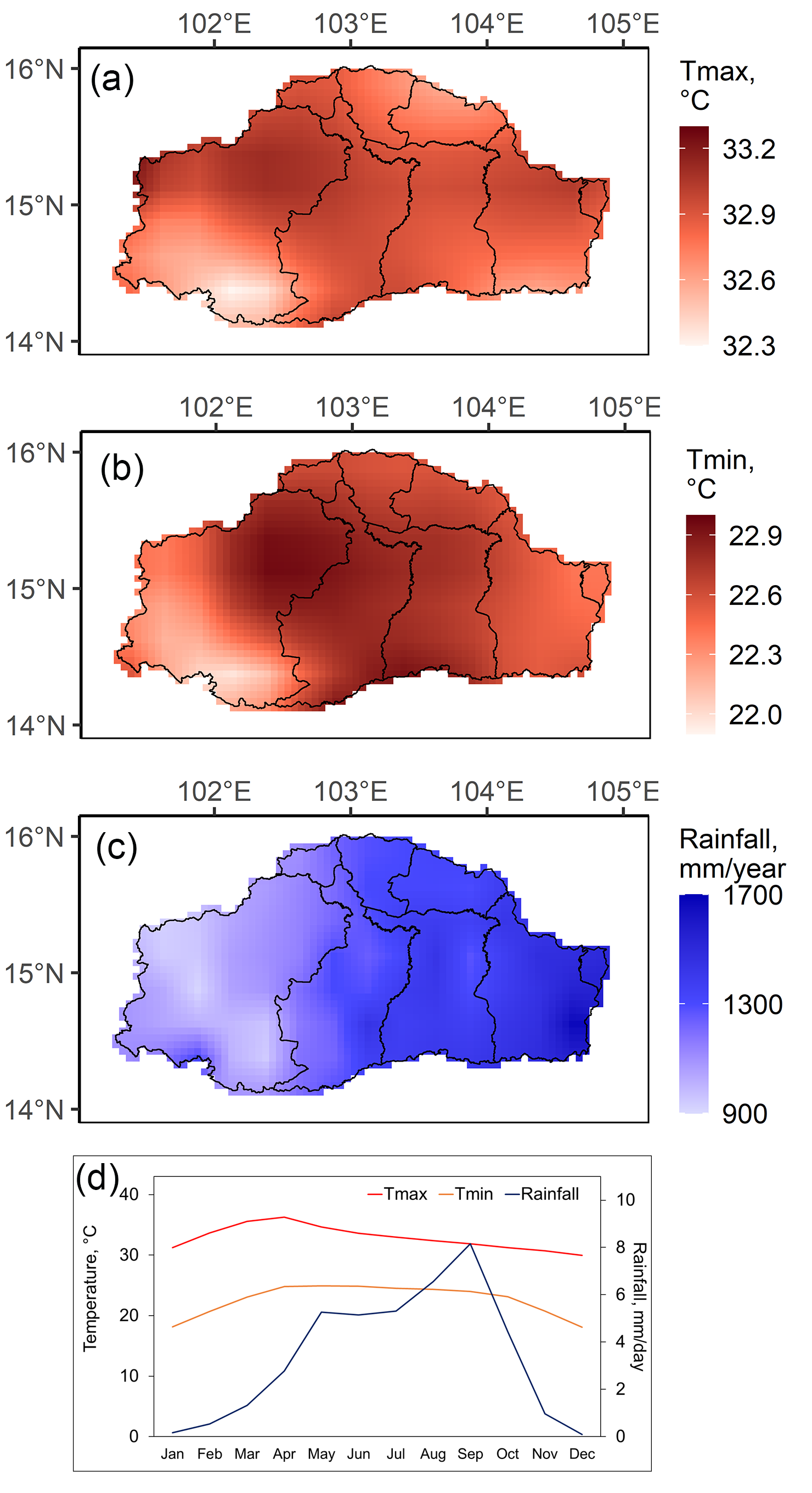


**Figure S2.** Observed climate during the baseline period (1981-2010) in the basin. Spatial pattern of the annual average (a) Tmax, (b) Tmin, (c) rainfall, and (d) basin averaged monthly values. All sub-plots are created using ggplot2 library in RStudio 2022.07.2+576 version.


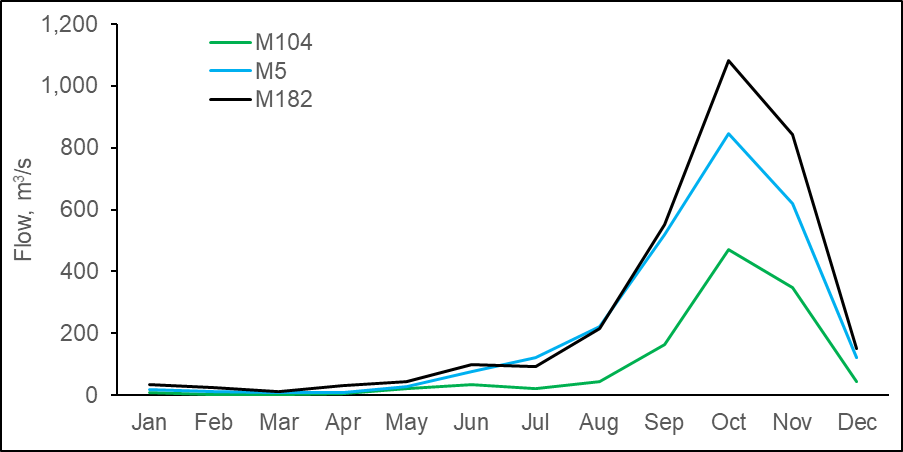


**Figure S3.** Monthly average flow at M104, M5, and M182 stations in the Mun river basin during the baseline period of 1981-2010. The figure is created in Microsoft 365.


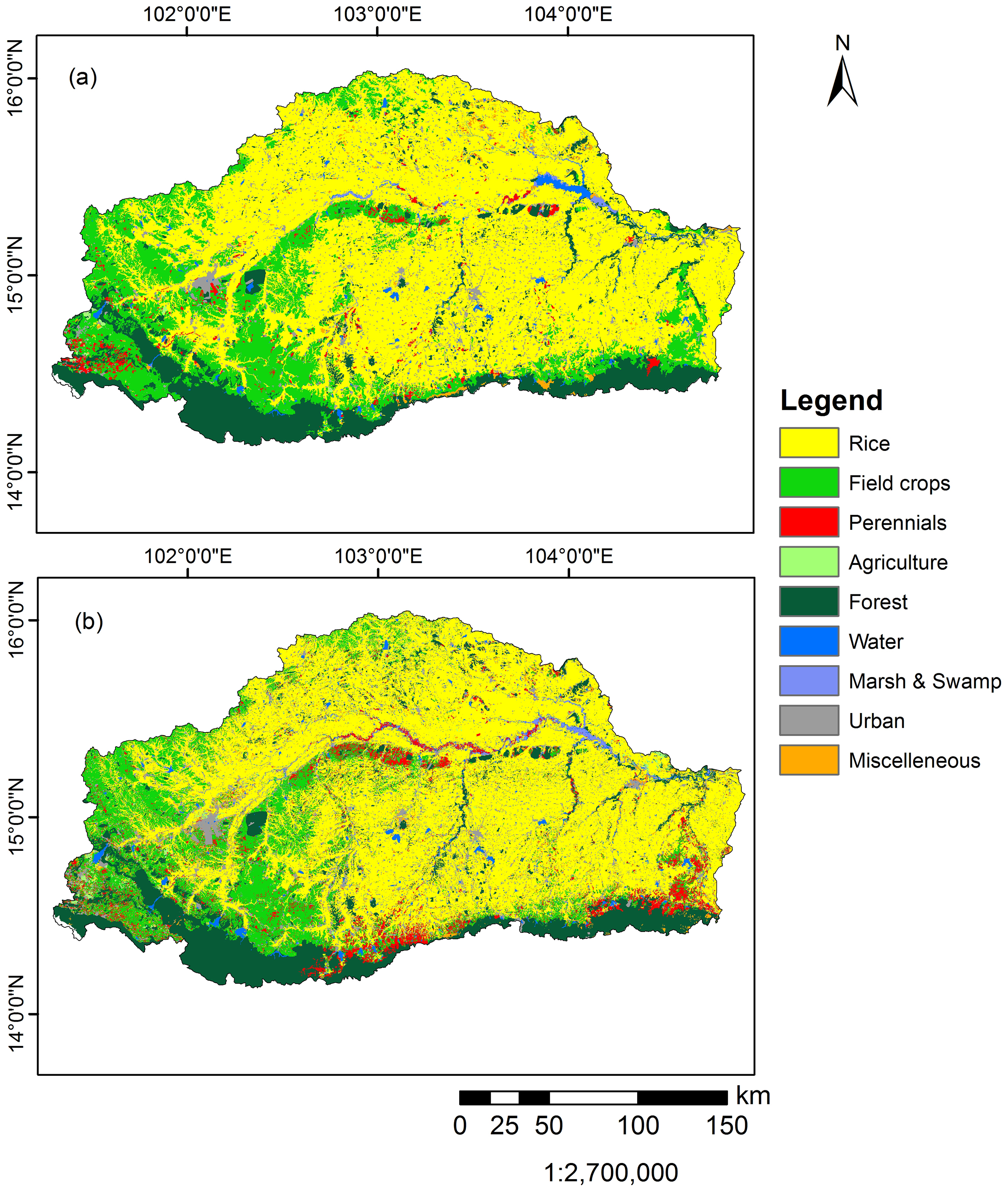


**Figure S4.** Land-use map of the basin with 9 major classes. (a) Land-use of 2000 (Rice 60.2%; Field crops 18.2%; Forest 12.2%; Urban 4.8%; Perennials & Orchards 1.7%; Water bodies 1.5%; Other Agriculture 0.1%; Marsh & Swamp 0.4%; and Miscellaneous 0.9%) and (b) Land-use of 2008 (Rice 55.5%; Field crops 14.3%; Forest 12.5%; Urban 6.1%; Perennials & Orchards 4.9%; Water bodies 2.7%; Other Agriculture 0.9%; Marsh & Swamp 0.7%; and Miscellaneous 2.5%). The figure is created in ArcGIS Pro 3.1.0.


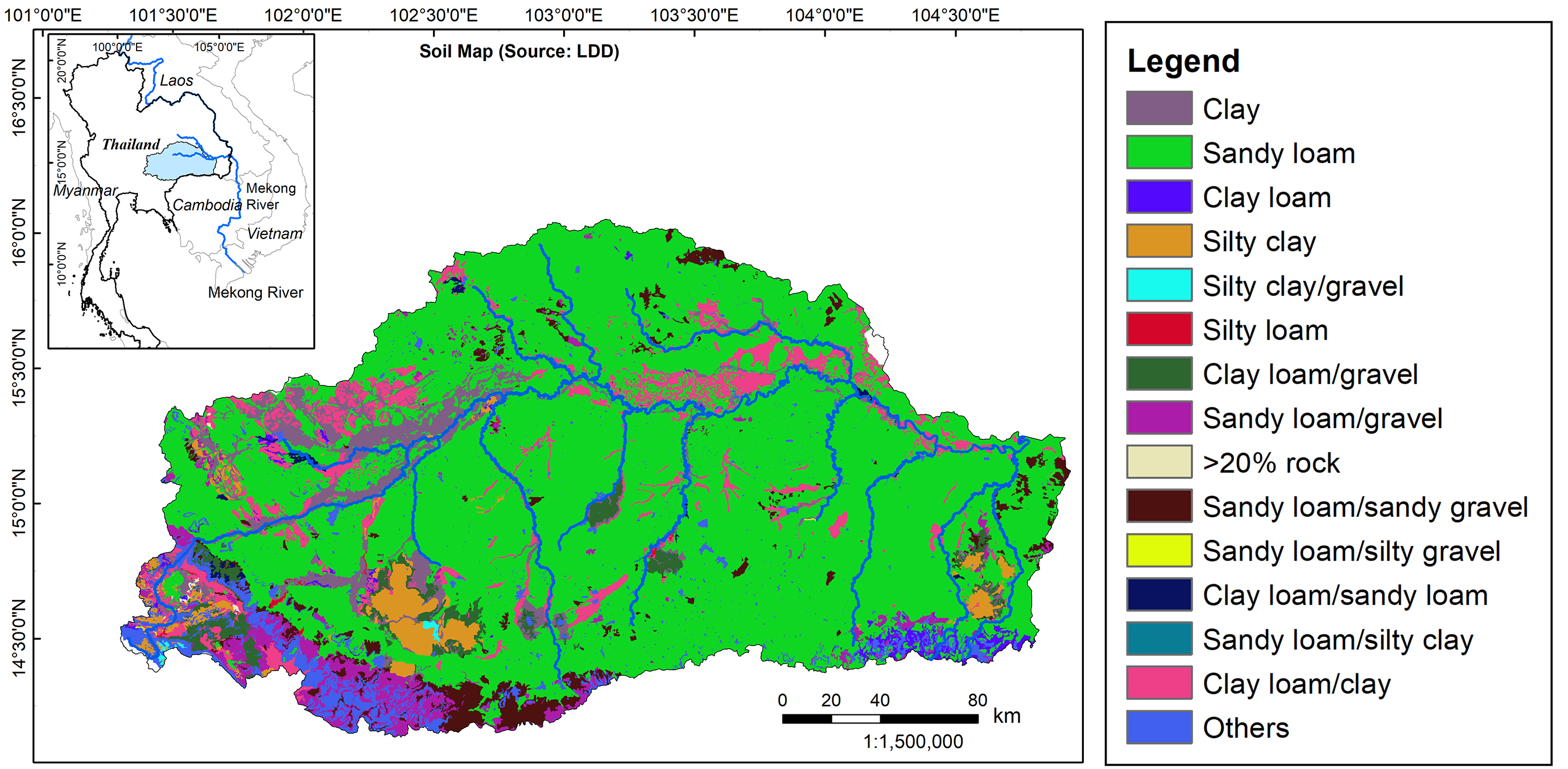


**Figure S5.** Soil map of the Mun river basin (Source: LDD, Thailand). The figure is created in ArcGIS Pro 3.1.0.


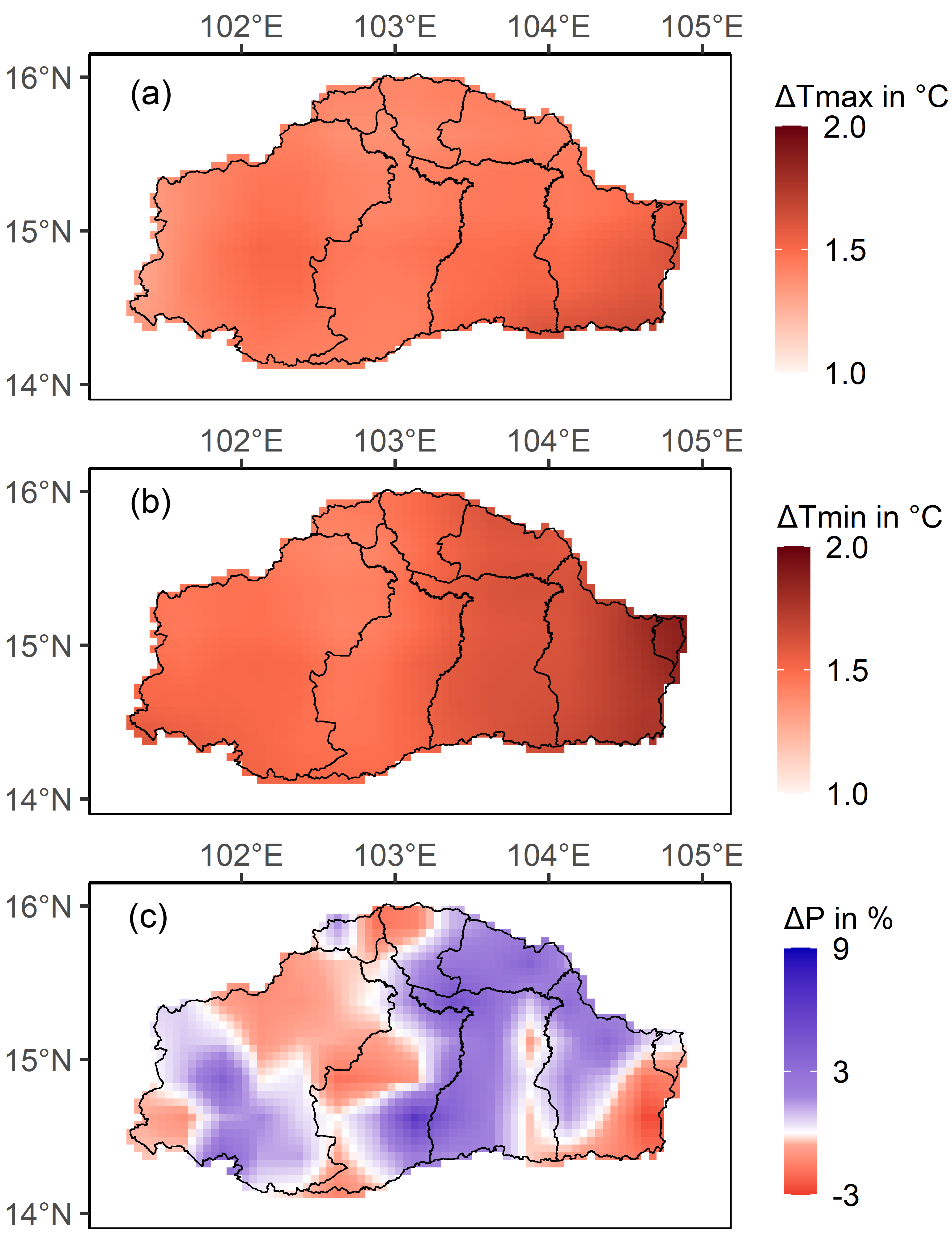


**Figure S6.** Climate change projections for the near-future (2021-2050). The spatial pattern of the projected changes in (a) Tmax, (b) Tmin, and (c) rainfall for CMIP6 –SSP5-8.5 using the multi-model average for the near-future period. Changes are with respect to the baseline period. All sub-plots are created using ggplot2 library in RStudio 2022.07.2+576 version.


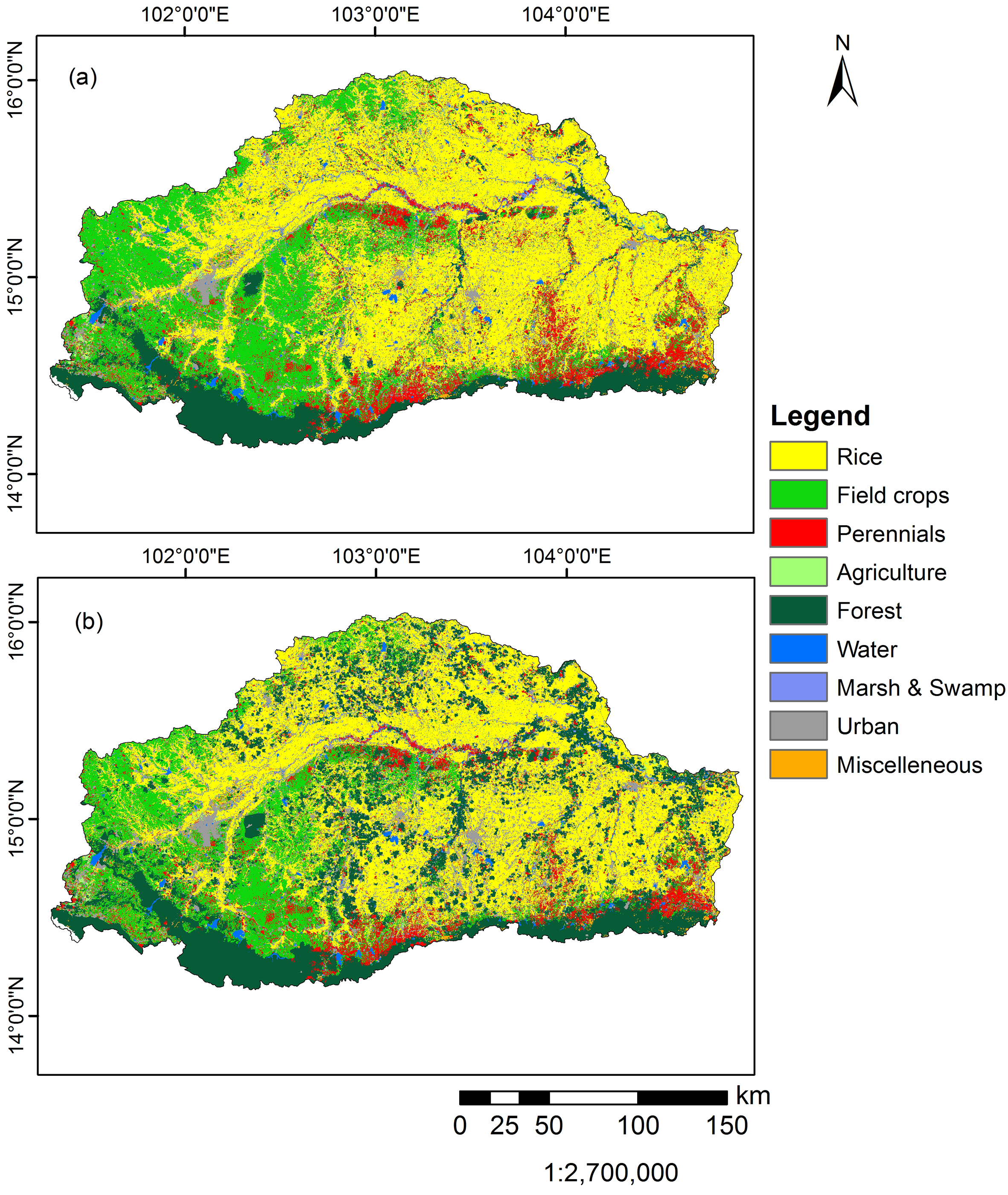


**Figure S7.** Land-use projections for the basin. (a) Projections under the Business as usual (BAU) scenario for 2050 and (b) Projection under the Combination of Forest Conservation and Urban Growth (CCU) scenario for 2050. The figure is created in ArcGIS Pro 3.1.0.

**Table S1.** Details of climate models from HighResMIPs of CMIP6 considered in the study.

| ID | Model Designation | Modeling Group | Atmospheric Resolution (lat × lon) | Number of vertical levels | Ensemble member |
| --- | --- | --- | --- | --- | --- |
| 1. | CNRM-CM6-1 | Centre National de Recherches Meteorologiques / Centre Europeen de Recherche et Formation Avancees en Calcul Scientifique | 1.4° x 1.4° | 91 | r1i1p1f2 |
| 2. | CNRM-CM6-1-HR | Centre National de Recherches Meteorologiques / Centre Europeen de Recherche et Formation Avancees en Calcul Scientifique | 0.5° x 0.5° | 91 | r1i1p1f2 |
| 3. | EC-Earth3P | EC-EARTH consortium | 0.7° x 0.7° | 91 | r1i1p2f1 |
| 4. | EC-Earth3P-HR | EC-EARTH consortium | 0.35° x 0.35° | 91 | r1i1p2f1 |
| 5. | HadGEM3-GC31-HH | UK Met Office Hadley Centre | 0.23° x 0.35° | 85 | r1i1p1f1 |
| 6. | HadGEM3-GC31-HM | UK Met Office Hadley Centre | 0.23° x 0.35° | 85 | r1i1p1f1 |
| 7. | HadGEM3-GC31-MM | UK Met Office Hadley Centre | 0.55° x 0.83° | 85 | r1i1p1f1 |
| 8. | HadGEM3-GC31-LL | UK Met Office Hadley Centre | 1.25° x 1.875° | 85 | r1i1p1f1 |

**Table S2.** Monthly and annual projected changes in Tmax, Tmin, and rainfall in the near-future period using the multi-model average.

|  |  | **Jan** | **Feb** | **Mar** | **Apr** | **May** | **Jun** | **Jul** | **Aug** | **Sep** | **Oct** | **Nov** | **Dec** | **Annual** |
| --- | --- | --- | --- | --- | --- | --- | --- | --- | --- | --- | --- | --- | --- | --- |
| **Baseline period** | **Max. Temp. (°C)** | 31.23 | 33.67 | 35.56 | 36.30 | 34.66 | 33.64 | 32.99 | 32.41 | 31.90 | 31.27 | 30.74 | 29.97 | 32.86 |
|  | **Min. Temp. (°C)** | 18.17 | 20.67 | 23.06 | 24.80 | 24.91 | 24.87 | 24.52 | 24.34 | 24.02 | 23.13 | 20.74 | 18.07 | 22.61 |
|  | **Rainfall (mm)** | 5.0 | 15.1 | 40.9 | 83.1 | 163.1 | 154.5 | 164.6 | 203.4 | 244.5 | 137.3 | 28.8 | 2.8 | 1,243 |
| **Projected Changes** | | | | | | | | | | | | | | |
| **Near-future period** | **Max. Temp. (°C)** | 1.10 | 1.23 | 1.45 | 1.52 | 1.44 | 1.07 | 1.20 | 1.27 | 1.12 | 1.22 | 1.45 | 1.42 | 1.29 |
|  | **Min. Temp. (°C)** | 1.45 | 1.43 | 1.29 | 1.26 | 1.40 | 1.28 | 1.50 | 1.41 | 0.96 | 1.11 | 1.65 | 1.77 | 1.37 |
|  | **Rainfall (%)** | 25.7% | -3.5% | -7.3% | -10.8% | -2.5% | 10.9% | 0.3% | -2.2% | 2.5% | 0.6% | 0.5% | 49.4% | 0.5% |
